# Supplementary material for: Unpacking the Lay Epidemiology of Cervical Cancer: A Focus Group Study on the Perceptions of Cervical Cancer and Its Prevention among Women Late for Screening in Norway
Source: Healthcare (Basel). 2023 May 15;11(10):1441. doi: 10.3390/healthcare11101441 (PMC10218218; doi:10.3390/healthcare11101441)
Supplement: Supplementary file 1 [file healthcare-11-01441-s001.zip › healthcare-2222043-supplementary.pdf]

VALID FROM 1.JULY 2018

Flow chart

for assessing liquid based cervical samples

Reason for taking a cervical sample?

The clinician must fill out reason for sampling and information relevant for evaluating the sample.

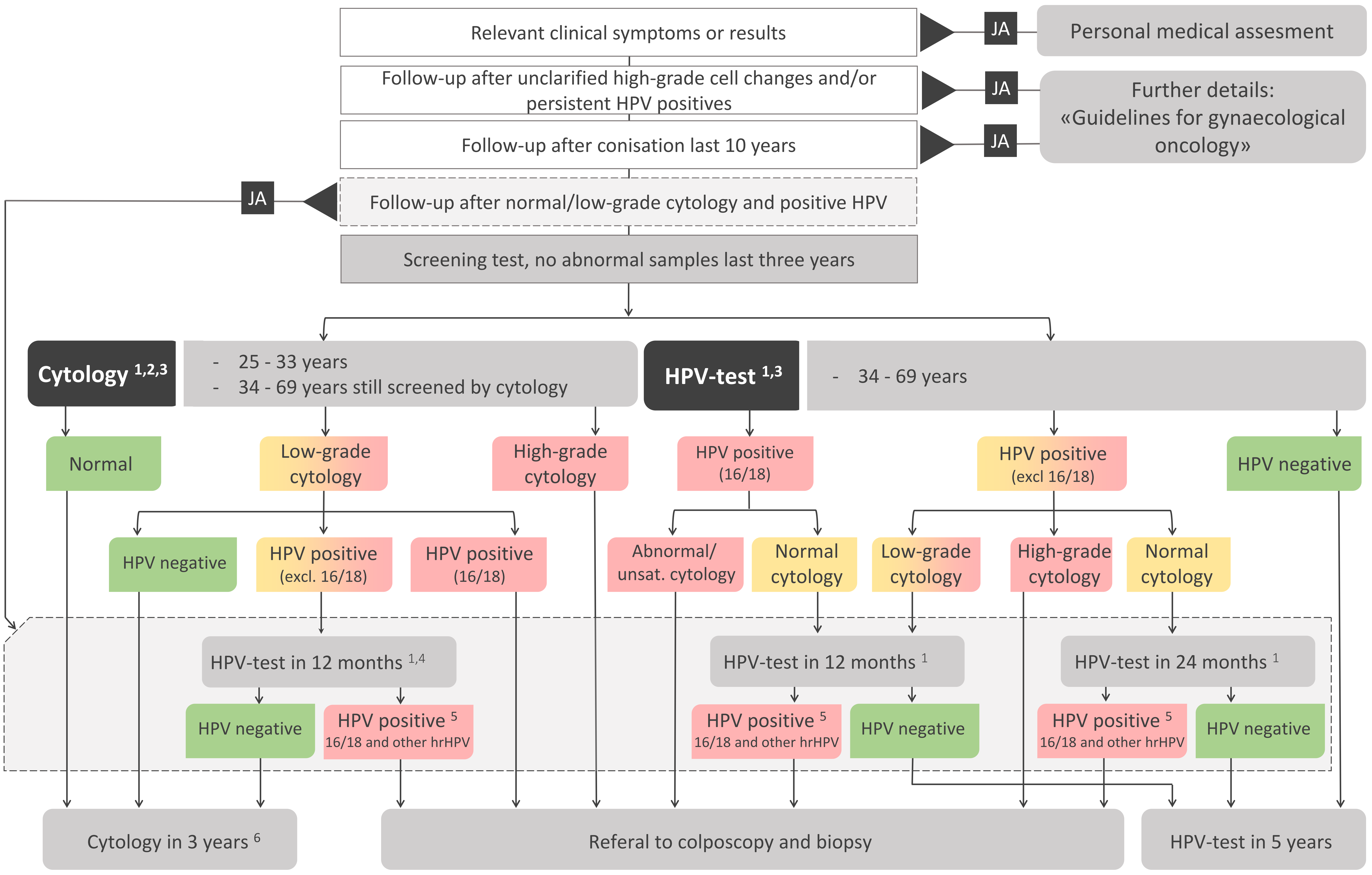

Explanations

Test result	
Recommendation	
Follow-up test	

Risk of high-grade cell lesions (CIN2+)

- Low (green)
- Intermediate (yellow)
- High (red)

HPV-test: Information about tests approved for use in the cervical cancer screening programme: https://www.kreftregisteret.no/krav-hpv-tester

Footnotes

1. If unsatisfactory test result (primary or reflex), new test in 1-3 months.
2. If two unsatisfactory cytology result, reflex-HPV.
3. For women aged 34 or more without previous cervical tests, or women without a cervical test last ten years, are recomended to have the sample cytologcial and HPV tested.
4. HPV-tests used in primary screening must be approved for use in the cervical cancer screening programme (https://www.kreftregisteret.no/krav-hpv-tester). Temporarily, cobas 4800 (Roche) may be used for analysing cervical samples on SurePath transport medium.
5. For HPV positive tests, cytology are to be carried out, but test results will not affect follow up. The result will be used by gynaecologist when carrying out colposcopic examination.
6. If the woman has turned 34 years and the region has implemented HPV primary screening, she should have a new HPV test in three years.

Low-grade cytology	ASCUS (atypical squamous cells of undetermined significance)
	LSIL (low-grade squamous intraepithelial lesion)
High-grade cytology	ASC-H (atypical squamous cells, cannot exclude a high-grade lesion)
	HSIL (high-grade squamous intraepithelial lesion)
	AGUS (atypical glandular cells of uncertain significance and atypical glandular cells of undetermined significance)
	ACIS (adenocarsinoma in situ)
	Ca (all types of cancer)
Abormal cytology	Low-grade or high-grade cytology
hrHPV	High-risk human papillomavirus
16/18	Genotype HPV16 and/or HPV18

Version 3, revised March 2020

Livmorhals programmet
